# Investigation of corneal topographic and densitometric properties of Wilson's disease patients with or without a Kayser-Fleischer ring

**DOI:** 10.1186/s40662-021-00231-9

**Published:** 2021-03-11

**Authors:** Mehmet Fuat Alakus, Mehtap Caglayan, Nazım Ekin, Hasan Oncul, Esref Arac, Umut Dag, Halit Diri

**Affiliations:** 1 Department of Ophthalmology, University of Health Sciences, Diyarbakir Gazi Yasargil Research and Training Hospital, Diyarbakır, Turkey; 2 Department of Internal Medicine, University of Health Sciences, Diyarbakir Gazi Yasargil Research and Training Hospital, Diyarbakır, Turkey

**Keywords:** Wilson, Kayser-Fleischer ring, Corneal densitometry

## Abstract

**Background:**

To investigate the topographic measurements and densitometry of corneas in Wilson’s disease (WD) patients with or without a Kayser-Fleischer ring (KF-r) compared to healthy individuals.

**Methods:**

This cross-sectional study included 20 WD patients without a KF-r (group I), 18 WD patients with a KF-r (group II), and 20 age-matched controls (group III). The Pentacam high resolution imaging system is used to determine corneal topographic measurements and densitometry.

**Results:**

Mean age for groups I, II and III was 25.40 ± 6.43 years (14–36 years), 25.38 ± 6.96 years (16–39 years), 23.60 ± 6.56 years (17–35 years), respectively (*P* = 0.623). There was no significant difference between the groups in terms of the anterior corneal densitometry values (*P* > 0.05), while the 6–10 mm and 10–12 mm mid stroma and the 2–6 mm, 6–10 mm, and 10–12 mm posterior corneal densitometry values in group II were significantly higher than those in groups I and III (for all values, *P* <  0.05). However, the 10–12 mm posterior corneal densitometry values in group I were also significantly higher than those in group III (*P* = 0.038). The central corneal thickness (CCT), thinnest corneal thickness (tCT), and corneal volume (CV) values in groups I and II were significantly lower than those in group III (for CCT values, *P* = 0.011 and *P* = 0.009; for tCT values, *P* = 0.010 and *P* = 0.005; for CV values, *P* = 0.043 and *P* = 0.029).

**Conclusion:**

In WD patients with a KF-r, corneal transparency decreased in the peripheral posterior and mid stromal corneal layers; for these patients, corneal transparency may be impaired not only in the peripheral cornea but also in the paracentral cornea.

## Background

Wilson’s disease (WD) is a hereditary autosomal recessive disease which is a hepatic copper metabolism dysfunction that results in copper accumulation in the hepatic tissues and extrahepatic tissues, such as the brain, cornea lenses, and kidneys [1–3]. WD can be clinically diagnosed via pathological examination and biochemically through urinary copper excretion and blood ceruloplasmin levels.

Ophthalmological manifestations of WD include the Kayser-Fleischer ring (KF-r), which is caused by the granular deposition of copper in the peripheral corneal Descemet membrane [2, 4]. The KF-r appears as a granular golden-greenish layer near the limbus. It first occurs at the top of the cornea and then inferiorly and finally appears circular, like a ring. The KF-r disappears after the treatment; this is therefore one way of determining the success of the treatment [2]. Sunflower cataracts, induced by deposits of copper in the middle of the lens, also disappear after treatment [2]. Other less common findings are night blindness, exotropic strabismus, optic neuritis, and pallor of the optic disc [5, 6].

The KF-r is not pathognomonic for WD but is used both as a diagnostic criterion and to monitor response to the therapy [7]. Therefore, clinicians request an ophthalmologist’s opinion in order to diagnose the patients who are suspected of having WD as a result of clinical and biochemical findings or evaluate the response to treatment. KF-r examination by ophthalmologists is generally carried out using a biomicroscopic slit lamp. Unfortunately, it is difficult to make a diagnosis by this method. Especially for the inexperienced clinicians or in case of arcus senilis which can prevent the visualization of the KF-r. Moreover, the KF-r was reported to be absent in approximately 50% of WD patients [8].

The Pentacam Heidelberg Corneal Topography is an optical imaging system that investigates anterior segment parameters, including the cornea, anterior chamber, and lenses. It can also be used to evaluate corneal and lens opacity [9, 10]. In this study, we compared the corneal topography and densitometry data obtained by Pentacam in healthy subjects and WD patients with and without a KF-r. As far as we know, there are only a few extant studies that have evaluated the densitometric properties of the cornea in WD and only one study that has evaluated its topographic features.

## Methods

This cross-sectional comparative study included patients diagnosed with WD as well as healthy subjects. This study was approved by the Ethics Committee of the Gazi Yaşargil Training and Research Hospital (Ref no: 2020/477) in accordance with the Declaration of Helsinki and all patients provided their written informed consent. The diagnoses of the WD patients were established by international diagnostic criteria and confirmed by genetic examination [11]. Fifty-four WD patients who were followed up in the gastroenterology clinic were examined ophthalmologically. There were 38 patients who met the inclusion criteria and consented to the study. The exclusion criteria were as follows: a spherical or cylindrical refractive error of > 1.50 D, corneal pathologies as dystrophies, corneal scars, keratitis, infections, keratoconus, a history of ocular trauma, uveitis glaucoma, a history of contact lens wear, a history of ocular surgery, use of eye drops during the preceding 6-month period, dry eye diseases, and any other systemic disease that might affect the cornea.

Group I included 20 eyes of 20 patients diagnosed with WD without a KF-r, group II included the 18 eyes of 18 patients diagnosed with WD with a KF-r, and group III included the 20 eyes of 20 healthy individuals. All 38 WD patients were taking D penicillamine combined with zinc salts therapy.

All participants were examined by four ophthalmologists and their data are recorded. Full biomicroscopic examinations of lens and fundus were performed using a slit lamp after tropicamide drop pupil dilatation. Intraocular pressure (IOP) was measured using a non-contact tonometer (Topcon CT-1P, Tokyo, Japan). Corneal endothelial cell density (CECD) values were measured using specular microscopy (Topcon Corporation, Tokyo, Japan) to detect endothelial insufficiency. Schirmer I test and tear break-up times (TBUTs) were noted to evaluate dry eye because both endothelial dysfunction and dry eye can affect corneal topography and densitometry values.

The Pentacam high resolution (HR) (Oculus optik Germate GmbH Wetzlar, Germany) which is a non-invasive optical system was used to investigate anterior segment parameters, such as the cornea, anterior chamber, and lenses. This imaging system uses a rotating Scheimpflug camera to produce anterior and posterior corneal topographic maps, corneal pachymetry, and three-dimensional analyses of the anterior chamber [9, 10]. Pentacam HR measurements of the patients are taken between 10 am and 12 noon 1 day after the examination so that corneal topography and densitometry values were not affected by diurnal variation, corneal staining, and IOP measurements. Flat keratometry (K1), steep keratometry (K2), maximum keratometry (Kmax), central corneal thickness (CCT), thinnest corneal thickness (tCT), and corneal volume (CV) values were recorded. Backward light scattering was measured using Scheimpflug tomography to evaluate changes in corneal transparency. The camera captures 25 single-slit images in two seconds while rotating around the eye from 0 to 180 degrees. All measurements were performed by the same experienced operator in the same room; dim-lighting was used.

**Fig. 1:**
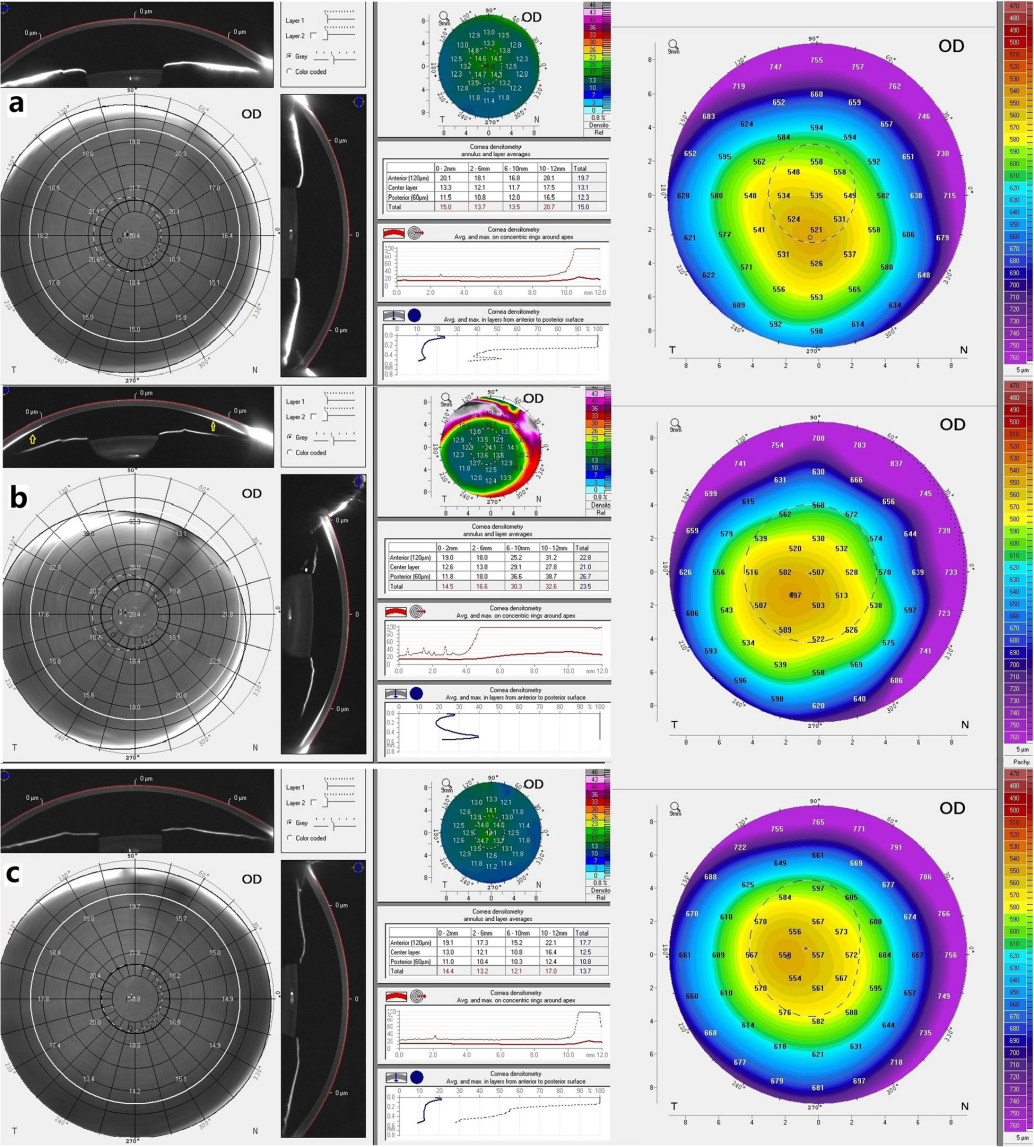
Pentacam HR-Scheimpflug images. **a** Image of a Wilson case without Kayser Fleischer ring. **b** Image of a Wilson case with Kayser Fleischer ring. c, Image of a healthy case

Corneal densitometry values were obtained according to a previously published program method [12]. The program automatically locates the corneal apex and analyses the area around the apex to a diameter of 12 mm [12, 13]. Depending on the degree of light scatter, the Pentacam HR quantifies the density of the cornea on a scale of 0–100 grayscale units (GSU), with 0 indicating no light scatter or no corneal haze and 100 indicating a totally opaque cornea. Local densitometric analyses were performed by dividing the 12 mm diameter areas into four concentric radial zones, available as software presets. The central zone had a diameter of 2 mm, centred on the apex. The second zone annuli extended from 2 mm to 6 mm in diameter, the third zone from 6 mm to 10 mm, and the final zone from 10 mm to 12 mm in diameter. The densitometry outputs were provided according to the corneal depths of the anterior, mid stroma, and posterior layers of the corneas. The anterior layers corresponded to the anterior 120 mm sections of the cornea, whereas the posterior layers corresponded to the posterior 60 mm sections of the cornea. The mid stromal (centre) corneal layers were defined by subtracting the anterior and posterior layers from the total corneal thickness (Fig. 1).

### Statistical analysis

Statistical data were analysed using the Statistical Package for the Social Sciences (SPSS) 20.00 program (SPSS Inc., Chicago, IL). Normality of the data was evaluated using the Shapiro Wilk test. Only the measurements of the right eye were used for statistical analysis. Statistical data were defined as the mean ± the standard deviation (SD) for numerical variables and as numbers for categorical variables. For comparisons between the three groups, the one-way ANOVA test with a Bonferroni Post Hoc test was used. Categorical variables were compared by using the Chi-squared test. Statistically, a *P* value less than 0.05 was considered significant. The power of the study was found to be 0.83 (calculated using the G-power software).

## Results

The mean ages of the groups were 25.40 ± 6.43 years (14–36 years), 25.38 ± 6.96 years (16–39 years), 23.60 ± 6.56 years (17–35 years) for groups I, II and III, respectively, and the groups were similar in terms of age and gender (*P* = 0.623 and *P* = 0.819, respectively). The demographic, clinical and ophthalmological examination findings of the cases in the groups are summarized in Table 1.

**Table 1:** The demographic and clinical features in groups

Variables	Group I (n = 20)	Group II (n = 18)	Group III (n = 20)	P
Age (years)	25.40 ± 6.43 (14–36)	25.38 ± 6.96 (16–39)	23.60 ± 6.56 (17–35)	0.623^*^
Gender (F/M)	9F/11M	9F/9M	11F/9M	0.819^†^
IOP (mm/Hg)	14.95 ± 3.05 (10–19)	13.83 ± 1.65 (10–16)	13.55 ± 2.21 (10–17)	0.161^*^
SE (D)	−0.07 ± 0.42 (− 0.60–0.51)	−0.10 ± 0.99 (−1.33–1.34)	−0.06 ± 0.78 (− 0.67–0.97)	0.105^*^
Schirmer (mm)	21.00 ± 5.78 (10–35)	20.61 ± 7.62 (12–35)	22.70 ± 7.02 (14–35)	0.604^*^
BUT (sec)	12.85 ± 3.23 (10–19)	12.38 ± 1.61 (11–18)	13.00 ± 2.33 (10–17)	0.741^*^
CECD (cell/mm^2^)	2599 ± 215 (2258–2895)	2652 ± 240 (2330–3001)	2539 ± 227 (2312–2998)	0.286^*^

Group I: Wilson’s disease without Kayser-Fleischer ring; Group II: Wilson’s disease with Kayser-Fleischer ring; Group III: Healthy individuals; *F=* female; *M=* male; *SE=* spherical equivalent; *IOP=* intraocular pressure; *BUT=* break-up time; *CECD=* corneal endothelial cell density; *n=* number of eyes

*: One-way ANOVA

†: Chi-squared test

While the K1, K2, and Kmax values were similar between the three groups (*P* > 0.05 for all values), the CCT, tCT, and CV values were significantly different between the groups (*P* = 0.003, *P* = 0.002 and *P* = 0.037, respectively). Groups I and II values were significantly lower than those for group III (for all values, *P* <  0.05; Table 2).

**Table 2:** Comparison of topographic values in groups

Variables	Group I (n = 20)	Group II (n = 18)	Group III (n = 20)	P*	Bonferroni Post Hoc Test
K1 (D)	43.04 ± 1.08 (41.50–44.70)	42.47 ± 0.81 (41.60–43.70)	42.76 ± 1.08 (41.10–44.00)	0.231	
K2 (D)	44.16 ± 1.11 (42.50–46)	43.22 ± 1.19 (41.60–45.50)	43.78 ± 1.30 (41.30–45.90)	0.063	
Kmax (D)	44.82 ± 1.26 (42.70–46.60)	43.94 ± 1.01 (42.60–45.80)	44.19 ± 1.34 (41.70–46.70)	0.081	
CCT (μm)	517.00 ± 34.62 (478–571)	515.44 ± 25.91 (489–557)	545.70 ± 28.54 (500–599)	0.003	I–III: 0.011 II–III: 0.009
tCT (μm)	510.55 ± 34.95 (467–552)	507.38 ± 25.72 (480–550)	539.90 ± 28.98 (495–589)	0.002	I–III: 0.010 II–III: 0.005
CV (mm^3^)	58.32 ± 3.94 (53.70–64.80)	58.51 ± 3.64 (52.30–67.70)	60.58 ± 2.95 (56.40–67.40)	0.037	I–III: 0.043 II–III: 0.029

Group I: Wilson’s disease without Kayser-Fleischer ring; Group II: Wilson’s disease with Kayser-Fleischer ring; Group III: Healthy individuals; *K1=* flat keratometry; *K2=* steep keratometry; *Kmax=* maximum keratometry; *D=* dioptre; *CCT=* central corneal thickness; *tCT=* thinnest corneal thickness; *CV=* corneal volume; *n=* number of eyes

*: One-way ANOVA

When corneal densitometry values were assessed, there was no significant difference between the groups in terms of the anterior corneal densitometry values ​​(*P* > 0.05 for all values). However, the midstroma and posterior corneal densitometry values were significantly different between the three groups, except for the 0–2 mm and 2–6 mm mid stroma and the 0–2 mm posterior corneal densitometry values ​​(*P* <  0.05 for all values, Table 3). Mean values of 6–10 mm and 10–12 mm mid stroma in group II were 15.53 ± 6.57 GSU (10.10–29.10 GSU) and 20.42 ± 5.43 GSU (13.90–31.80 GSU), respectively, and these values were significantly higher than groups I and III (*P* values for 6–10 mm I – II: 0.002, II – III: 0.004; for 10–12 mm I – II: 0.031, II – III: 0.014, respectively). The posterior corneal densitometry values of 2–6 mm, 6–10 mm, and 10–12 mm in group II were 11.53 ± 3.45 GSU (8.90–18.00 GSU), 34.37 ± 4.23 GSU (23.20–41.40 GSU) and 35.10 ± 4.09 GSU (24.40–42.40 GSU), respectively. Similarly, the values in group II were significantly higher than groups I and III (*P* values for 2–6 mm, I – II: 0.035, II – III: 0.038, for 6–10 mm I – II: < 0.001, II – III: < 0.001, for 10–12 mm I – II: < 0.001, II – III: < 0.001, respectively) (Table 3). In addition, the 10–12 mm posterior corneal densitometry value in group I was also significantly higher than those in group III (*P* = 0.038) (Table 3).

**Table 3:** Comparison of anterior, centre, and posterior corneal densitometry values

Variables	Group I (*n* = 20)	Group II (*n* = 18)	Group III (n = 20)	P^*^	Bonferroni Post Hoc Test
Anterior (60 μm)					
0–2 mm	19.70 ± 1.52 (17.30–22.50)	19.19 ± 1.69 (17.10–22.30)	18.91 ± 2.18 (15.30–21.80)	0.391	
2–6 mm	17.59 ± 1.24 (15.40–19.70)	17.00 ± 1.26 (15.40–19.90)	17.29 ± 1.33 (15.00–18.90)	0.380	
6–10 mm	17.16 ± 2.25 (14.20–21.70)	17.83 ± 3.50 (14.80–27.20)	17.20 ± 2.54 (14.40–25.90)	0.714	
10–12 mm	28.76 ± 7.02 (19.00–37.20)	29.27 ± 4.77 (18–36.50)	27.44 ± 88 (14.70–37.00)	0.075	
Mid stroma					6–10 mm
0–2 mm	12.60 ± 0.98 (11.10–14.80)	12.30 ± 0.77 (11.50–14.50)	12.85 ± 0.70 (11.80–14.00)	0.132	I–II: 0.002 II–III: 0.004
2–6 mm	11.27 ± 0.78 (9.80–12.60)	11.41 ± 1.02 (10.50–14.80)	11.51 ± 0.54 (10.30–12.30)	0.636	10–12 mm
6–10 mm	11.03 ± 1.14 (9.30–14.40)	15.53 ± 6.57 (10.10–29.10)	11.32 ± 1.33 (9.90–14.90)	0.001	I–II: 0.031 II–III: 0.014
10–12 mm	18.96 ± 2.86 (14.60–23.60)	20.42 ± 5.43 (13.90–31.80)	16.21 ± 4.57 (8.30–26.60)	0.015	
Posterior (120 μm)					2–6 mm
0–2 mm	10.59 ± 0.80 (9.40–12.50)	10.40 ± 0.76 (9.60–11.80)	10.70 ± 0.87 (9.10–12.50)	0.534	I–II: 0.035 II–III: 0.038
2–6 mm	9.82 ± 0.72 (8.90–11.20)	11.53 ± 3.45 (8.90–18.00)	10.00 ± 0.73 (8.70–11.20)	0.023	6–10 mm
6–10 mm	12.32 ± 4.55 (9.30–24.90)	34.37 ± 4.23 (23.20–41.40)	10.51 ± 1.21 (8.70–13.60)	< 0.001	I–II: < 0.001 II–III: < 0.001
10–12 mm	16.68 ± 3.87 (11.70–25.30)	35.10 ± 4.09 (24.40–42.40)	13.83 ± 3.78 (7.60–21.60)	< 0.001	10–12 mm I–II: < 0.001 I–III: 0.038 II–III: < 0.001

Group I: Wilson’s disease without Kayser-Fleischer ring; Group II: Wilson’s disease with Kayser-Fleischer ring; Group III: Healthy individuals; n = number of eyes

*: One-way ANOVA

## Discussion

In the current study, it was established that the eyes with both WD involvement and a KF-r, had significantly increased centre and posterior corneal densitometry values in the peripheral paracentral cornea. In addition, the corneal thickness and volume values were significantly lower in WD patients with and without a KF-r than in healthy individuals.

Corneal densitometry is a parameter related to the transparency of the cornea and is affected by histological changes to the cornea. Analysis of corneal densitometry has gained importance since the introduction of the Pentacam densitometry program. Using this program, densitometry values, which are the elements of corneal optic quality in the cornea, can be obtained quickly, repeatedly, and non-invasively [14]. Regular spacing of the collagen fibres and extracellular matrix, balanced keratocyte components, and levels of corneal light backscatter may be observed to be impaired in corneal transparency [15]. Corneal densitometry is often affected by ocular surface diseases, such as keratitis, dry eye, and pseudoexfoliation syndrome, as well as some systemic disease like diabetes mellitus, mucopolysaccharidosis, and Fabry disease [12, 13, 15–18].

The KF-r can be detected via biomicroscopic examination, and gonioscopic examination, but success of its detection depends on the clinician’s experience with WD. Hence, in recent years, many researchers have investigated the KF-r by using anterior segment OCT, in vivo corneal microscopy, and Scheimpflug imaging systems, which provide more objectivity in corneal examinations. Ceresara et al. have investigated KF-r using confocal microscopy and found copper deposits in the peripheral cornea in 75% of their WD patients, whereas by slit lamp, copper deposits were seen in only 25% of their WD patients [19]. Zhao et al. have shown that there were abnormal patterns in the peripheral Descemet membranes using confocal microscopy in all 52 WD patients with a KF-r [20]. Recent studies have also shown that anterior segment OCT results can be used for detecting KF-r in WD patients. The KF-r was imaged in greyscale hyper-reflective layers along the corneal periphery using anterior segment OCT in WD patients [21, 22].

Although there are a limited number of studies in the literature, the topographic and densitometric features of the cornea can also be examined by using devices containing the Scheimpflug imaging system, which has high ease of use and repeatability. Telinius et al. have reported that it was possible to detect KF-r with very high sensitivity and specificity by using Scheimpflug imaging [23]. The authors have reported that in cases whose KF-r were prominent in biomicroscopic examinations, Pentacam HR showed KF-r as hyperreflective pre-Descemet bands in the periphery. They have also reported that the presence or absence of the KF-r cannot be differentiated using Pentacam imaging by a built-in densitometry module for the corneal examination of cases without prominent KF-r. In those cases, the authors have found that the mean values for the densitometry of the anterior and posterior parts of the cornea and the ratio between them have to be calculated. They therefore state that the diagnostic accuracy of Pentacam’s built-in densitometry module was poor and a more detailed analysis of images should therefore be pursued with the use of the ImageJ software. The peak posterior value was significantly higher in patients with a KF-r compared to that of patients without a KF-r and that of healthy controls. The study included 10 patients with a KF-r accumulation in the inferior part of the cornea and also examined 10–12 mm peripheral anterior and posterior densitometry values [23].

In another recent study regarding corneal densitometry in WD, Doguizi et al. have found that in paediatric WD patients without a KF-r, the corneal densitometry values were higher in the posterior 6–10 mm and 10–12 mm zones than those of control subjects [24]. There was no statistical difference in the corneal densitometry values of the other zones and layers. They showed that in patients without a KF-r, copper accumulation alters corneal densitometry. The most important limitation of this study was the exclusion of a group of patients with a KF-r [24].

In our study, we compared the densitometry of WD patients and normal healthy adults. There was no statistical difference in anterior corneal densitometry between WD patients with or without a KF-r and the control group. However, we found that the densitometry values of WD patients with a KF-r were higher in the centre 6–10 mm and 10–12 mm zones and the posterior 2–6 mm, 6–10 mm, and 10–12 mm zones than those without a Kf-r and of the control group. The KF-r group had the highest densitometry value for all posterior zones except the 0–2 mm zone. Interestingly, for the posterior 2–6 mm zone, densitometry values were higher in WD with Kf-r patients; this has not been reported until now. The increase in densitometry at 2–6 mm indicates that the central cornea can be affected during the course of WD with Kf-r. In this study, mean corneal densitometer values in the posterior 6–10 mm and 10–12 mm corneal zones were higher in WD patients without Kf-r compared to the control group. These results are congruent with those reported by Doğuizi et al. However, in our study, this difference was statistically significant only for the posterior 10–12 mm corneal zone. The densitometry module of Scheimpflug imaging systems can guide clinicians in the detection of Kf-r in WD patients. The fact that posterior peripheral corneal densitometry values are found to be higher than the healthy group even in cases where Kf-r cannot be detected biomicroscopically may suggest that there are corneal accumulations in these cases even in the early stage. Therefore, the KF-r can be used as a clinical parameter to monitor patients undergoing therapy [7]. Although its reduction is not necessarily well correlated with clinical improvement, its reappearance may indicate the ineffectiveness of the treatment [25]. Thus, Scheimpflug densitometric imaging systems can provide clinicians with more objective data in treatment monitoring.

In this study, corneal topographic values were also examined. We observed that CCT, tCT, and CV values were significantly lower in WD patients with and without a KF-r versus the healthy group. Kara et al. found that CCT was thinner in WD patients when compared to healthy subjects and in addition, WD patients with a KF-r had thinner CCT than WD patients without a KF-r [26]. The CCT value is important in terms of possible keratorefractive surgery in WD patients, helping surgeons to be detect WD patients. Copper accumulation can be toxic in tissues as copper may reduce the level of glutathione, an important antioxidant. When oxidative stress increases, the levels of reactive oxygen species also increase, and thus lead to extracellular matrix degeneration and stromal thinning in the cornea [27, 28].

Our study is the largest to have investigated corneal densitometry in WD patients, but is not without limitations. Firstly, in vivo confocal microscopic analyses with the Scheimpflug imaging system could definitely provide more valuable information, allowing a better understanding of corneal densitometry and changes in corneal thickness. The only method that can show real-time images of corneal layers at the cellular level in high resolution is confocal microscopes. Therefore, a cellular imaging system is needed to explain the increased peripheral corneal densitometry values especially in non-Kf-r Wilson patients. Another limitation is that we did not examine the role of corneal densitometry for responses to treatment.

## Conclusion

In conclusion, this study showed that the WD patients with a KF-r have decreased corneal transparency in their peripheral posterior and midstromal corneal layers. The transparency was also affected in the central and paracentral cornea. Pentacam HR can be used for the diagnosis and treatment in WD patients as well as the evaluation of the optical quality in cornea. These findings should be supported by further studies with a larger patient population.

## Data Availability

The datasets used and/or analysed during the current study are available from the corresponding author on reasonable request.

## Abbreviations

WD: Wilson’s Disease
KF-r: Kayser-Fleischer ring
OCT: Optical coherence tomography
HR: High resolution
IOP: Intraocular pressure
CECD: Corneal endothelial cell density
TBUTs: Tear break-up times
K1: Flat keratometry
K2: Steep keratometry
Kmax: Maximum keratometry
CCT: Central corneal thickness
tCT: Thinnest corneal thickness
CV: Corneal volume
GSU: Grayscale units
SD: Standard deviation
